# Dependence of Interaction Free Energy between Solutes on an External Electrostatic Field

**DOI:** 10.3390/ijms140714408

**Published:** 2013-07-11

**Authors:** Pei-Kun Yang

**Affiliations:** Department of Biomedical Engineering, I-SHOU University, Kaohsiung 84001, Taiwan; E-Mail: peikun@isu.edu.tw; Tel.: +886-7-6151-100 (ext. 7477); Fax: +886-7-6155-150

**Keywords:** athermal effect, potential of mean force, protein conformation, protein-protein/ligand interactions

## Abstract

To explore the athermal effect of an external electrostatic field on the stabilities of protein conformations and the binding affinities of protein-protein/ligand interactions, the dependences of the polar and hydrophobic interactions on the external electrostatic field, −***E***^ext^, were studied using molecular dynamics (MD) simulations. By decomposing ***E***^ext^ into, along, and perpendicular to the direction formed by the two solutes, the effect of ***E***^ext^ on the interactions between these two solutes can be estimated based on the effects from these two components. ***E***^ext^ was applied along the direction of the electric dipole formed by two solutes with opposite charges. The attractive interaction free energy between these two solutes decreased for solutes treated as point charges. In contrast, the attractive interaction free energy between these two solutes increased, as observed by MD simulations, for *E*^ext^ = 40 or 60 MV/cm. ***E***^ext^ was applied perpendicular to the direction of the electric dipole formed by these two solutes. The attractive interaction free energy was increased for *E*^ext^ = 100 MV/cm as a result of dielectric saturation. The force on the solutes along the direction of ***E***^ext^ computed from MD simulations was greater than that estimated from a continuum solvent in which the solutes were treated as point charges. To explore the hydrophobic interactions, ***E***^ext^ was applied to a water cluster containing two neutral solutes. The repulsive force between these solutes was decreased/increased for ***E***^ext^ along/perpendicular to the direction of the electric dipole formed by these two solutes.

## 1. Introduction

Among the four fundamental interactions, the electrostatic force dominates non-covalent bond interactions between atoms in biomolecules such as proteins, DNA, and RNA. An external electrostatic field may alter the electrostatic interactions among atoms in proteins, and consequently change the stabilities of protein conformations or the binding affinities of protein-protein/ligand interactions. Changing the stability of the protein conformation may change the protein activity, and changing the binding affinities of the protein-protein/ligand interactions may change the regulation of the signal transduction network in cells or the expression of proteins. Although these effects may not cause diseases immediately, they may increase the possibility of diseases developing. Further, electromagnetic radiation is used to kill bacteria for food preservation. Potential problems have been studied [1,2] using protein experiments [3–7], cellular experiments [8–11], animal experiments [12], public health data [13], and computer simulations [14–16]. The results did not verify whether electromagnetic radiation is harmful to humans.

In most cases, the interactions in biomolecules or among biomolecules are regarded as the sum of the interactions among the atoms in the biomolecules. Understanding the interactions among atoms in biomolecules is therefore helpful in exploring problems such as the stability of protein conformations and the binding affinities of protein-protein/ligand interactions. In addition, understanding the effects of an external electrostatic field, −***E***^ext^, on the interactions among atoms in biomolecules is helpful in exploring the effects of the external electric field on the stabilities of protein conformations or the binding affinities of protein-protein/ligand interactions. To explore the effects of ***E***^ext^ on the mean force between two charged atoms in water, ***E***^ext^ was decomposed into two components: one along the electric dipole formed by these two charged atoms, and the other perpendicular to this electric dipole. The effect of ***E***^ext^ on the mean force between the two charged atoms in water was estimated by summing the effects on the mean force from these two components.

Most biomolecules exist in aqueous environments, and the interactions among atoms in water differ from those in vacuum. The solvent effect is significant for the stabilities of protein conformations [17–21] and the binding affinities of protein-protein/ligand interactions [22–25]. Numerous strategies have been developed for computing the solvation free energy [26–36]. By treating the charged atom as a point charge, the effect of ***E***^ext^ on the interactions among charged atoms in water can be quickly estimated using Coulomb’s law. However, strategies using molecular dynamic (MD) simulations with explicit solvent models afford more accurate results.

For one or two charged atoms in a spherical water cluster, the dependence of the electric dipole of TIP3P water on the net electrostatic field at the oxygen atom of TIP3P water, and the dependence of the radial distribution function of TIP3P water on the mean force at the oxygen atom of TIP3P water, have been explored [33,37,38]. In this project, the source code of the Charmm package [39] was modified, and it was verified that it can be applied to an external electrostatic field. The external electrostatic field was applied to a simulation system containing one charged atom or two charged atoms in vacuum; the accelerations, velocities, and positions of atoms from MD simulations using the modified Charmm package were consistent with those obtained from analytical solutions (data not shown). The external electrostatic field along the *x* or *y* direction (*E*_X_^ext^, *E*_Y_^ext^) was applied to a pure water cluster, and the relation between the dipole moment of TIP3P water and the net electrostatic field at the oxygen atom of TIP3P water was consistent with the results obtained in previous works [37,38]. The external electrostatic field along the *x* or *y* direction (*E*_X_^ext^, *E*_Y_^ext^) was applied to a water cluster containing one or two charged atoms, and the dependences of the mean force and the potential of mean force (PMF) between the charged solutes on ***E***^ext^ were studied using MD simulations [40]. The differences between the mean force estimated from a continuum solvent and that computed using MD simulations were discussed.

## 2. Results/Discussion

### 2.1. Dependence of *p* on *E*^ext^

**Figure 1:**
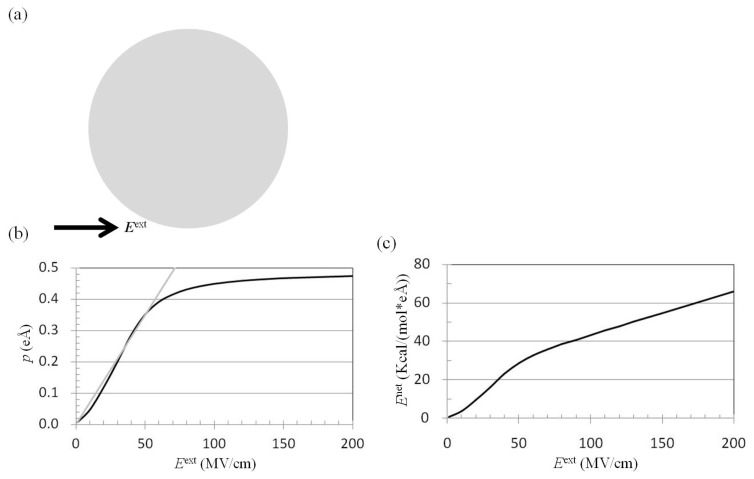
Dependences of *p* and *E*^net^ on *E*^ext^. (**a**) The external electrostatic field, −***E***^ext^, was applied to a water cluster with a radius of 20 Å containing 1119 TIP3P [41] water molecules; (**b**) The time-averaged dipole moment per water molecule, −***p*** (black line), was computed from the trajectories of molecular dynamics (MD) simulations. The *p* (gray line) was plotted as 0.007 eÅ/(MV/cm) *E^ext^ (MV/cm); (**c**) The time-averaged electrostatic field at the oxygen atom of TIP3P water, −***E***^net^ (black line), was computed from the trajectories of MD simulations.

On applying ***E***^ext^ to a water cluster containing solutes, the water molecules were polarized by the ***E***^ext^. The electric force on the atoms of the solute from ***E***^ext^ could be shielded by the polarized water molecules. To explore the effect of the electric dipole per water molecule, −***p***, polarized by ***E***^ext^, ***E***^ext^ was applied to the water cluster (Figure 1a), and ***p*** and the net electric field on the water molecule, −***E***^net^ were computed from the trajectories of MD simulations using (1) and (3), respectively. The results showed that ***p*** was proportional to ***E***^ext^ in the region |*E*^ext^| < 50 MV/cm. The ratio of *p* to *E*^ext^ was approximately 0.007 eÅ/(MV/cm), as *p*_E_ = 0.007 eÅ/(MV/cm) * *E*^ext^ (MV/cm). The *p* (black line) from MD simulations was compared with *p*_E_ (gray line) (Figure 1b). With regard to the relationship between *E*^net^ and *E*^ext^, *E*^net^ was proportional to *E*^ext^. The proportionality constant was larger in the region |*E*^ext^| < 50 MV/cm than in the region |*E*^ext^| > 50 MV/cm (Figure 1c). Based on these results, the external electrostatic field along the *x* direction, −*E*_X_^ext^ = 40, 60, or 100 MV/cm, or the external electrostatic field along the *y* direction, −*E*_Y_^ext^ = 50 or 100 MV/cm, was applied to a water cluster containing two solutes to compute the mean force and the PMF on the solute *S*_2_.

**Figure 2:**
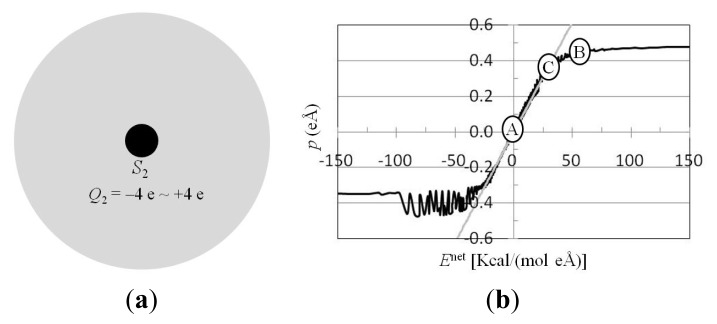
Dependence of *p* on *E*^net^. (**a**) A solute with a charge of −4.0 e, −3.0 e, −2.0 e, −1.0 e, −0.8 e, −0.6 e, −0.4 e, −0.2 e, +0.2 e, +0.4 e, +0.6 e, +0.8 e, +1.0 e, +2.0 e, +3.0 e, or +4.0 e was at the center of a spherical water cluster of radius 20 Å containing 1118 TIP3P [41] water molecules. The van der Waals parameters of the solute assigned were the same as those for the oxygen atom of TIP3P water with ɛ = −0.1521 kcal/mol and *R*_min_/2 = 1.7682 Å; (**b**) The ***p***(*Q*_2_, *r*) and ***E***^net^(*Q*_2_, *r*) were computed from the trajectories of MD simulations. For |*Q*_2_| ≤ 4 e and *r* ≤ 10 Å, dependence of ***p*** on ***E***^net^ was shown (black line). Because the TIP3P water model is not a point dipole moment, ***p*** does not only depend on ***E***^net^. The *p*_E_ (gray line) was plotted as 0.0124 (mol·e^2^·Å^2^)/kcal * *E*^net^ [kcal/(mol·eÅ)].

The dependence of ***p*** on the charged solute and ***E***^net^ has been extensively studied [37,38]. A solute with a charge of −4.0 e, −3.0 e, −2.0 e, −1.0 e, −0.8 e, −0.6 e, −0.4 e, −0.2 e, +0.2 e, +0.4 e, +0.6 e, +0.8 e, +1.0 e, +2.0 e, +3.0 e, or +4.0 e was at the center of a spherical water cluster (Figure 2a), and ***p*** and ***E***^net^ were computed from the trajectories of MD simulations (Figure 2b). The solvent molecular polarizability ɛ_0_γ_mol_ was defined as d*p*/d*E*^net^. Δ*p* was proportional to Δ*E*^net^ in the region |*E*^net^| < 25 kcal/(mol·eÅ) (1 MV/cm = 0.231 kcal/(mol·eÅ)), and ɛ_0_γ_mol_ = 0.0124 (mol·e^2^·Å^2^)/kcal (or γ_mol_ = 51.7 Å^3^[37]). For *E*^net^ > 50 kcal/(mol·eÅ), the net dipole of TIP3P is towards the direction of ***E***^net^, and the value of *p* approaches +0.49 eÅ. For *E*^net^ < −100 kcal/(mol eÅ), one of the hydrogen atoms was toward the anion, and the value of *p* approaches −0.35 eÅ. In the region −100 kcal/(mol·eÅ) < *E*^net^ < −50 kcal/(mol·eÅ), the net dipole of TIP3P or one of the hydrogen atoms could be toward the anion, so the value of *p* depends not only on *E*^net^, but also depends on the solute charge and position in calculating ***E***^net^. The values of *p* therefore varied between −0.49 eÅ and −0.35 eÅ.

The *p* from *E*^ext^ was compared with that from the charged solute. The ***p*** of water molecules polarized by ***E***^ext^ (MV/cm) (Figure 1a) was compared with that polarized by a solute with charge *Q*(e) (Figure 2a). For example, when *E*^ext^ = 50 MV/cm was applied to a pure water cluster, *p* = 0.35 eÅ (Figure 1b), which is 70% of the permanent electric dipole moment of TIP3P water. For the solute with a charge of +1.0 e in water, and the same van der Waals (vdW) parameters as the oxygen atom of TIP3P water, *p* at the first peak of the radial distribution function, solute distance 2.8 Å, was 0.35 eÅ [37,38]. The subconclusion is that *p* polarized by *E*^ext^ = 50 MV/cm was similar to *p* at the first peak of the radial distribution function surrounding the solute with a charge of +1.0 e.

*E*^ext^ shifts the equilibrium position of the *p* − *E*^net^ curve of the water molecule, and could reduce the electric shielding effect between charged atoms in macromolecules. Consider a macromolecule such as a protein in water solvent; the solvent molecules are polarized by the charged atoms in the macromolecules, and the polarized molecules shield the electrostatic interactions between the charged atoms in the macromolecules. The solvent molecular polarizability, γ_mol_, describes the electric shielding effect between charged particles in dielectrics. In the case of no applied ***E***^ext^, the equilibrium position is at position A (Figure 2b). The Δ***p*** is proportional to Δ***E***^net^ in the region |*E*^net^| < 25 kcal/(mol eÅ), and ɛ_0_γ_mol_ = 0.0124 (mol·e^2^·Å^2^)/kcal. Applying ***E***^ext^ = 200 MV/cm to the water cluster leads to *E*^net^ = 66 kcal/(mol·eÅ). The equilibrium position is shifted to position B (Figure 2b). The ɛ_0_γ_mol_ for computing the electric shielding effect between charged atoms in macromolecules is 0.0006 (mol·e^2^·Å^2^)/kcal. Applying ***E***^ext^ = 50 MV/cm to the water cluster leads to *E*^net^ = 29 kcal/(mol·eÅ). The equilibrium position is shifted to position C (Figure 2b). The γ_mol_ was 0.0007/0.0022 (mol·e^2^·Å^2^)/kcal in the direction of increasing/decreasing *p*. The subconclusion is that the electric shielding effect of water for computing the electric interactions between charged atoms in macromolecules could be reduced by *E*^ext^.

### 2.2. Dependence of *F*^net^(One_Atom) on *E*^ext^

**Figure 3:**
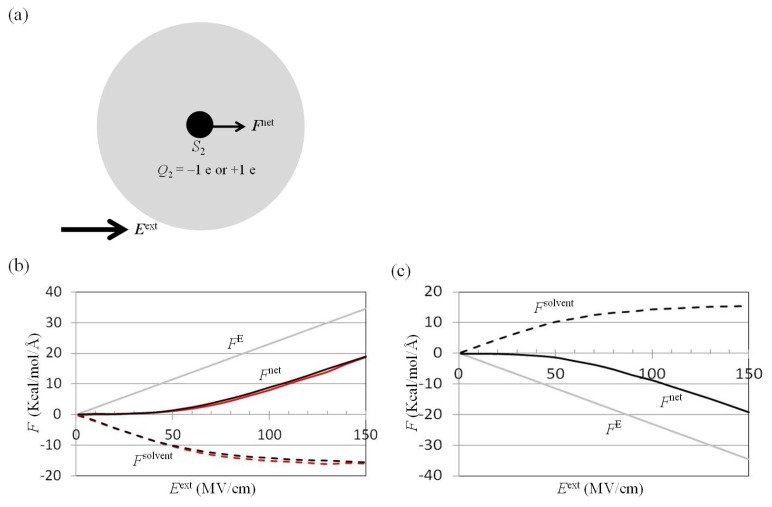
Dependence of *F*(one_atom) on *E*^ext^. (**a**) The external electrostatic field ***E***^ext^ was applied to a water cluster containing one charged solute *S*_2_. The solute *S*_2_ was at the center of a spherical water cluster of radius 20 or 25 Å containing 1118 or 2185 TIP3P [41] water molecules. To explore the general effect of ***E***^ext^ on the atoms in biomolecules, the van der Waals parameters of the solute were assigned to be the same as the oxygen atom of TIP3P water with ɛ = −0.1521 kcal/mol and *R*_min_/2 = 1.7682 Å. For *Q*_2_ = +1 e (**b**) or −1 e (**c**), the ensemble average force on the charged solute contributed by *E*^ext^ − *F*^E^ (gray line), the polarized water molecules, −***F***^solv^ (dashed line), and the net force −*F*^net^ (solid line) were computed from the trajectories of MD simulations with an amplitude of *E*^ext^ ranging from 0.1 to 150 MV/cm. The radii of the water clusters were 20 Å (black line) and 25 Å (red line), respectively.

To understand the effect of ***E***^ext^ on the force on the charged solute, ***E***^ext^ was applied to a water cluster of radius 20 Å, and the mean force on the solute *S*_2_ was computed using the trajectories of the MD simulations (Figure 3a). The net mean force, −***F***^net^, was contributed by the external electrostatic field −***F***^E^ and the polarized water molecules −***F***^solv^, using (9) and (10) [38,42]. The results showed that |*F*^net^| was small in the |*E*^ext^| < 50 MV/cm region because the ***F***^E^ force was almost balanced by the ***F***^solv^ force (Figure 3b). For |*E*^ext^| > 50 MV/cm, the dielectric water approached saturation, |*F*^solv^| increased slowly as *E*^ext^ increaed, and *d*|*F*^net^|/*dE*^ext^ approached a constant (Figure 3b). To understand the dependences of the ***F***^net^ and ***F***^solv^ forces on the radius of the water cluster, ***E***^ext^ was applied to a water cluster of radius 25 Å containing one solute with a charge of +1.0 e. The results showed that the ***F***^net^ and ***F***^solv^ computed from the water cluster of radius of 25 Å were similar to those obtained using a radius of 20 Å (Figure 3b).

The TIP3P water molecule contained one oxygen and two hydrogen atoms. The vdW radius of the oxygen atom, *R*_min_/2 = 1.7682 Å, was larger than that of the hydrogen atom, *R*_min_/2 = 0.2245 Å. For the cation in water, the oxygen atom of water was closer to the cation. In contrast, for the anion in water, the hydrogen atom of water was closer to the anion. The radial distribution function of the oxygen or hydrogen atoms surrounding the cation therefore differed from that of those surrounding the anion [37]. However, the amplitude of ***F***^net^ on the solute with a charge of +1 e was similar to that on the solute with a charge of −1 e (Figure 3c). This means that the ***F***^net^(one_atom; *E*^ext^) was independent of the sign of the charged solute.

### 2.3. Attractive Force between *S*_1_ and *S*_2_ Could Not Be Decreased by Applying an External Electrostatic Field along the Direction of the Electric Dipole Formed by *S*_1_ and *S*_2_

**Figure 4:**
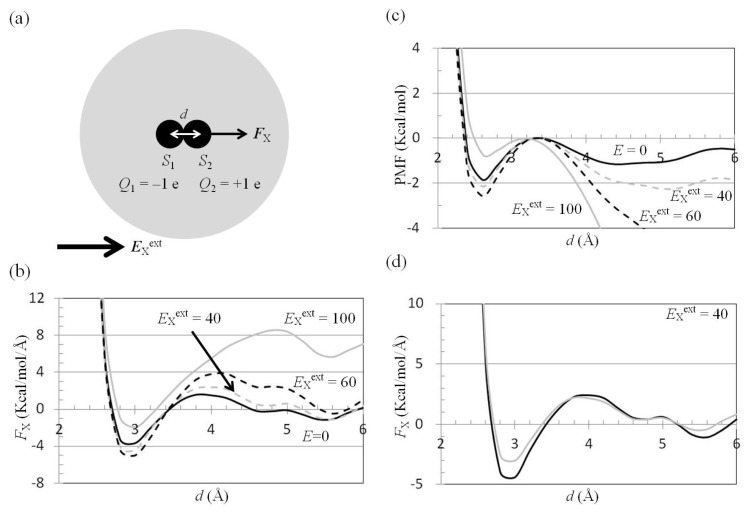

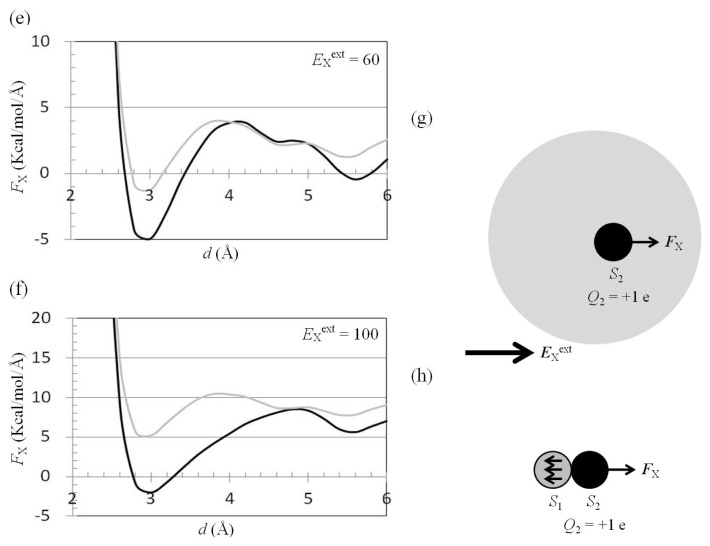
*F*_X_(two_atoms; *E*_X_^ext^) − *d* curves. (**a**) An external electrostatic field along the *x* direction, *E*_X_^ext^, was applied to a water cluster containing *S*_1_ and *S*_2_ solutes. The solute *S*_1_, with charges *Q*_1_ = −1 e positioned at (−*d*/2, 0, 0), and the solute *S*_2_, with charges *Q*_2_ = +1 e positioned at (+*d*/2, 0, 0), were in a spherical water cluster of radius 20 Å containing 1117 TIP3P [41] water molecules. The distance between the *S*_1_ and *S*_2_ solutes was from 2 to 8 Å. The vdW parameters of the *S*_1_ and *S*_2_ solutes were assigned to be the same as those of the oxygen atom of TIP3P water with ɛ = −0.1521 kcal/mol and *R*_min_/2 = 1.7682 Å; (**b**) The mean forces along the *x* direction, −*F*_X_, on the solute *S*_2_ were computed from the trajectories of MD simulations with external electrostatic fields *E*_X_^ext^ = 0 (solid black), 40 MV/cm (dashed gray), 60 MV/cm (dashed black), and 100 MV/cm (solid gray), respectively; (**c**) The potentials of mean force were computed from the mean forces in (b). *F*_X_(two_atoms; *E*_X_^ext^) (black line) was compared with *F*_X_^est^(two_atoms; *E*_X_^ext^) (gray line) for *E*_X_^ext^ = 40 MV/cm (**d**), 60 MV/cm (**e**), and 100 MV/cm (**f**); (**g**) *E*_X_^ext^ was applied to a water cluster containing the *S*_2_ solute. (**h**) The force on *S*_2_ was contributed by the polarized water molecules in the space occupied by *S*_1_. The dielectric polarization in the space occupied by *S*_1_ in (**h**) was the reverse of the dielectric polarization in the space occupied by *S*_1_ in (**g**).

Polar interactions, such as those between hydrogen bond donors and acceptors, play a significant role in stabilizing protein conformations and protein-protein/ligand complex structures. On applying ***E***^ext^ to macromolecules containing polar interactions, the electric dipole formed by the two atoms with opposite charges, *S*_1_ and the *S*_2_, prefers to align in the direction of ***E***^ext^, to reduce the potential energy, and ***E***^ext^ pulls *S*_1_ and pushes *S*_2_ along the direction of ***E***^ext^ (Figure 4a). The attractive force and the attractive interaction free energy between *S*_1_ and *S*_2_ decreased if *S*_1_ and *S*_2_ were treated as point charges in continuum dielectrics.

For *S*_1_ and *S*_2_ exposed to water, the external electrostatic field along the *x* direction, −*E*_X_^ext^, was applied to the water cluster containing *S*_1_ and *S*_2_ solutes (Figure 4a). The mean force on *S*_2_ along the *x* direction, −*F*_X_(two_atoms; *E*_X_^ext^), was computed using the trajectories of the MD simulations. The results showed that *F*_X_(two_atoms; *E*_X_^ext^ = 0) was attractive (negative) in the 2.7 Å < *d* < 3.4 Å region, and the minimum value of *F*_X_(two_atoms; *E*_X_^ext^ = 0) was −3.7 kcal/(mol·Å) at the position *d* = 3.0 Å (Figure 4b). We also applied *E*_X_^ext^ = 40 or 60 MV/cm to a water cluster containing one solute with a charge of +1 e, and the mean force *F*_X_(one_atom; *E*_X_^ext^) on the solute with a charge of +1 e was positive (Figure 3a). Was the attractive force on the solute *S*_2_ in Figure 4a decreased by application of the external electrostatic field? The results from the MD simulations showed that *F*_X_(two_atoms; *E*_X_^ext^ = 40 or 60 MV/cm) was more attractive than *F*_X_(two_atoms; *E*_X_^ext^ = 0) in the region 2.7 Å < *d* < 3.4 Å. The minimum values of *F*_X_(two_atoms; *E*_X_^ext^) at position *d* = 3.0 Å were −4.4 and −5.0 kcal/(mol Å) for *E*_X_^ext^ = 40 and 60 MV/cm, respectively (Figure 4b).

The PMF, −*P*_MF_(two_atoms; *E*_X_^ext^ = 40 or 60 MV/cm), was calculated by integration of the mean force ***F***_X_ from infinity. For comparison of the energies needed to escape the first well of *P*_MF_(two_atoms; *E*_X_^ext^ = 40 or 60 MV/cm), the second peak of *P*_MF_(two_atoms; *E*_X_^ext^), was set at zero. The results showed that the depth of the first well of *P*_MF_(two_atoms; *E*_X_^ext^ = 40 or 60 MV/cm) was deeper than that of *P*_MF_(two_atoms; *E*_X_^ext^ = 0) (Figure 4c).

Treating *S*_1_ and *S*_2_ as point charges, *F*_X_(two_atoms; *E*_X_^ext^) was estimated by summation of *F*_X_(two_atoms; *E*_X_^ext^ = 0) (Figure 4b) and *F*_X_^net^(one_atom; *E*_X_^ext^) (Figure 3b). *F*_X_^est^(two_atoms; *E*_X_^ext^) was the sum of *F*_X_(two_atoms; *E*_X_^ext^ = 0) in Figure 4b and *F*_X_^net^(one_atom; *E*_X_^ext^) in Figure 3b. The results showed that *F*_X_^est^(two_atoms; *E*_X_^ext^) was larger than *F*_X_(two_atoms; *E*_X_^ext^), especially in the *d* < 3.4, 3.8, and 4.4 Å regions for *E*_X_ = 40, 60, and 100 MV/cm, respectively (Figure 4d−f). This is because no water molecules can be polarized in the space occupied by *S*_1_. If *S*_1_ occupies the space, *F*_X_(two_atoms; *E*_X_^ext^) should be estimated by summation of *F*^X^(two_atoms; *E*_X_^ext^ = 0) (Figure 4b), *F*_X_^net^(one_atom; *E*_X_^ext^) (Figure 4g), and *F*_X_(excluded_solvent; *E*_X_^ext^) (Figure 4h), based on the superposition principle. *F*_X_(excluded_solvent; *E*_X_^ext^) is the force on solute *S*_2_ contributed by the water in the space occupied by *S*_1_. The dielectric polarization in the space occupied by *S*_1_ in Figure 4h was the reverse of the dielectric polarization in the space occupied by *S*_1_ in Figure 4g. *F*_X_(excluded_solvent; *E*_X_^ext^) was along the −*x* direction, therefore *F*_X_^est^(two_atoms; *E*_X_^ext^) was larger than *F*_X_(two_atoms; *E*_X_^ext^).

### 2.4. Attractive Force between *S*_1_ and *S*_2_ Was Unchanged and Increased by Applying *E*_Y_^ext^ = 50 MV/cm and 100 MV/cm, Respectively

**Figure 5:**
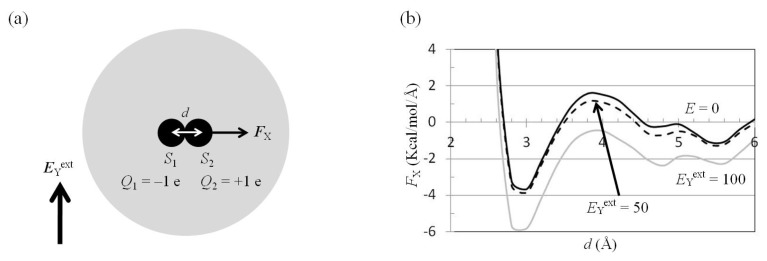
*F*_X_(two_atoms; *E*_Y_^ext^) − *d* curves. (**a**) An external electrostatic field along the *y* direction, *E*_Y_^ext^, was applied to a water cluster containing *S*_1_ and *S*_2_ solutes. The others were the same as those in Figure 4a; (**b**) The mean forces along the *x* direction, −*F*_X_, on solute *S*_2_ were computed from the trajectories of MD simulations with external electrostatic fields *E*_Y_ = 0 (solid black), 50 MV/cm (dashed black), and 100 MV/cm (solid gray).

The external electrostatic field along the *y* direction, −*E*_Y_^ext^, was applied to a water cluster containing *S*_1_ and *S*_2_ solutes (Figure 5a). The mean force on *S*_2_ along the *x* direction, −*F*_X_(two_atoms; *E*_Y_^ext^), was computed using the trajectories of the MD simulations. The results showed that *F*_X_(two_atoms; *E*_Y_^ext^ = 50 MV/cm) was similar to *F*_X_(two_atoms; *E*_ext_ = 0), but *F*_X_(two_atoms; *E*_Y_^ext^ = 100 MV/cm) was less than *F*_X_(two_atoms; *E*_ext_ = 0) (Figure 5b). The difference between *F*_X_(two_atoms; *E*_Y_^ext^ = 50 MV/cm) and *F*_X_(two_atoms; *E*_ext_ = 0) at the position of the first minimum of *F*_X_(two_atoms; *E*_ext_ = 0) was 0.3 kcal/(mol Å), and the difference between *F*_X_(two_atoms; *E*_Y_^ext^ = 100 MV/cm) and *F*_X_(two_atoms; *E*_ext_ = 0) at the position of the first minimum of *F*_X_(two_atoms; *E*_ext_ = 0) was 2.2 kcal/(mol·Å) (Figure 5b).

*E*_ext_ was applied to the charged atom in the water cluster (Figure 2a), the force on the charged atom was along the direction of ***E***_ext_, and the force perpendicular to direction of ***E***_ext_ was zero. *S*_1_ and *S*_2_ were treated as point charges. ***E***_ext_ was applied perpendicular to the direction of the electric dipole formed by *S*_1_ and *S*_2_; the force on *S*_1_ and *S*_2_ from ***E***_ext_ was along the direction of ***E***_ext_, and the attractive force and the interaction potential energy between *S*_1_ and *S*_2_ were unchanged. When *E*_ext_ = 100 MV/cm was applied perpendicular to the direction of the electric dipole formed by these two atoms (Figure 5a), the attractive force between *S*_1_ and *S*_2_ increased (Figure 5b), and the interaction free energy also increased, as observed from MD simulations. This is because polarization of the water molecules was saturated on application of *E*_Y_^ext^ = 100 MV/cm. The saturated water molecule is hard to polarize further by *S*_1_ and *S*_2_. The dielectric shielding effect between *S*_1_ and *S*_2_ was therefore reduced.

### 2.5. *F*_Y_(Two_Atoms; *E*_Y_^ext^) Was Greater than *F*_Y_(one_atom; *E*_Y_^ext^), Especially When *d* Was Small

**Figure 6:**
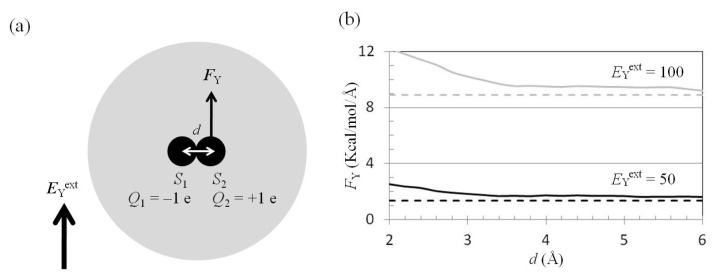

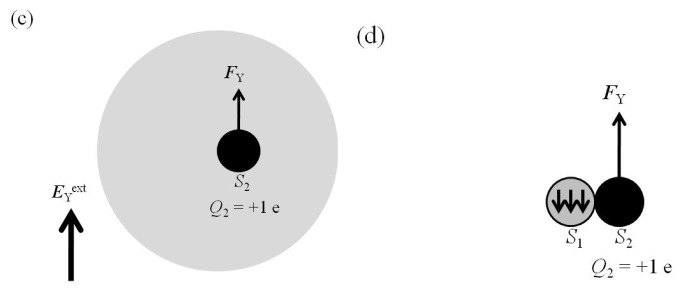
*F*_Y_(two_atoms; *E*_Y_^ext^) − *d* curves. (**a**) The external electrostatic field along the *y* direction, −*E*_Y_^ext^, was applied to a water cluster containing two charged solutes. The other parameters were the same as those in Figure 4a; (**b**) The mean forces along the *y* direction, −*F*_Y_, on solute *S*_2_ were computed from the trajectories of MD simulations with external electrostatic fields *E*_Y_ = 50 MV/cm (solid black) and 100 MV/cm (solid gray). *F*_Y_^net^(two_atoms; *E*_Y_^ext^) was compared to the force on solute *S*_2_ in Figure 3a with external electrostatic fields *E*_Y_ = 50 MV/cm (dashed black) and 100 MV/cm (dashed gray); (**c**) *E*_Y_^ext^ was applied to a water cluster containing *S*_2_; (**d**) The force on *S*_2_ was contributed by the polarized water molecules in the space occupied by *S*_1_. The dielectric polarization in the space occupied by *S*_1_ in (**d**) was the reverse of the dielectric polarization in the space occupied by *S*_1_ in (**c**).

*E*_Y_^ext^ was applied to a water cluster containing two charged solutes (Figure 6a); the mean force on *S*_2_ along the *y* direction, −*F*_Y_(two_atoms; *E*_Y_^ext^), was computed using the trajectories of the MD simulations. The results showed that *F*_Y_(two_atoms; *E*_Y_^ext^ = 50 or 100 MV/cm) was similar to *F*_Y_(one_atom; *E*_Y_^ext^ = 50 or 100 MV/cm), except in the region *d* < 3.0 Å (Figure 6b). The differences between *F*_Y_(two_atoms; *E*_Y_^ext^) and *F*_Y_(one_atom; *E*_Y_^ext^) at *d* = 6 Å were 0.2 and 0.3 kcal/(mol Å) for *E*_Y_^ext^ = 50 and 100 MV/cm, respectively.

The force on *S*_2_ from *S*_1_ was along the −*x* direction. Treating *S*_1_ and *S*_2_ as point charges, *F*_Y_(two_atoms; *E*_Y_^ext^) should be the same as *F*_Y_(one_atom; *E*_Y_^ext^). However, the force on *S*_2_ in the water cluster containing two solutes was greater than the force on the charged solute *S*_2_ in the water cluster containing one solute (Figure 6b). This is because no water molecules in the space occupied by *S*_1_ can be polarized. *F*_Y_(two_atoms; *E*_Y_^ext^) should be estimated by summation of *F*_Y_(one_atom; *E*_Y_^ext^) (Figure 6c) and *F*_Y_(excluded_solvent; *E*_Y_^ext^) (Figure 6d), based on the superposition principle. *F*_Y_(excluded_solvent; *E*_Y_^ext^) is the force on solute *S*_2_ contributed by the water molecules in the region occupied by solute *S*_1_. The dielectric polarization in the space occupied by *S*_1_ in Figure 6d was the reverse of the dielectric polarization in the space occupied by *S*_1_ in Figure 6c. *F*_Y_(excluded_solvent; *E*_Y_^ext^) was along the +*y* direction (Figure 6d), therefore *F*_Y_(one_atom; *E*_Y_^ext^) was less than *F*_Y_(two_atoms; *E*_Y_^ext^).

### 2.6. Dependence of F_X_(Two_Neutral_Atoms) on *E*_X_^ext^ and *E*_Y_^ext^

**Figure 7:**
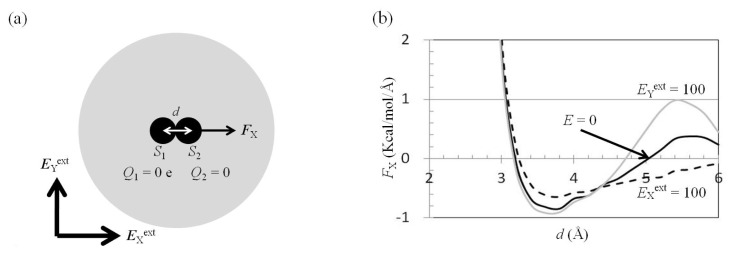

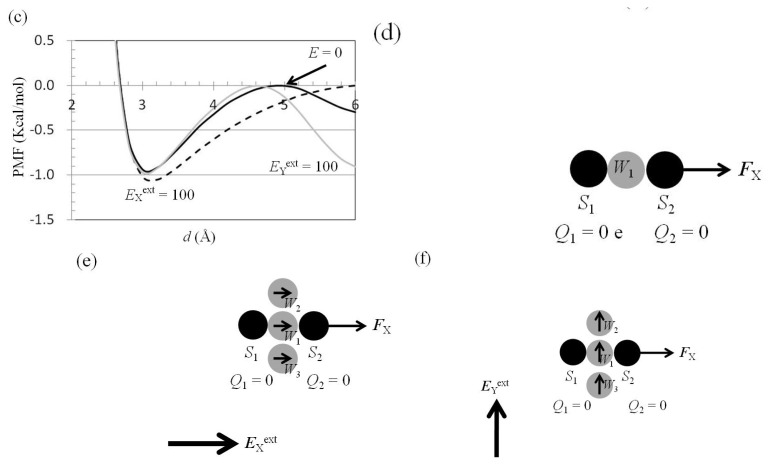
*F*_X_(two_neutral_atoms; *E*_X_^ext^/*E*_Y_^ext^)−*d* curves. (**a**) Two neutral solutes separated by a distance *d* along the *x* direction were in a TIP3P water cluster of radius 20 Å. An external electrostatic field along the *x* or *y* direction was applied to the water cluster. The other parameters were the same as those in Figure 4a; (**b**) The mean forces along the *x* direction, *F*_X_, on solute *S*_2_ were computed from the trajectories of MD simulations with *E*^ext^ = 0 (solid black), *E*_X_^ext^ = 100 MV/cm (dashed black), and *E*_Y_^ext^ = 100 MV/cm (solid gray); (**c**) The potentials of mean force were computed from the mean forces in (b); (**d**) For solutes *S*_1_ and *S*_2_ separated by a distance greater than 5.4 Å, the space between *S*_1_ and *S*_2_ can accommodate a water molecule, *W*_1_; (**e**) On applying *E*_X_^ext^ = 100 MV/cm to this water cluster, the water molecules will be polarized along the *x* direction. *W*_1_ will be pushed by the neighboring water molecules, *W*_2_ and *W*_3_; (**f**) On applying *E*_Y_^ext^ = 100 MV/cm to this water cluster, the water molecules will be polarized along the *y* direction. *W*_1_ will be attracted by the neighboring water molecules, *W*_2_ and *W*_3_.

Hydrophobic interactions play a significant role in the stabilities of protein conformations and the binding affinities of protein-protein/ligand interactions. The effect of an external electrostatic field on the mean force between two neutral solutes was explored. Consider two neutral solutes in a water cluster (Figure 7a). The mean force on *S*_2_, −*F*_X_(two_neutral_atoms; *E*^ext^), was computed using the trajectories of the MD simulations. The results showed that *F*_X_(two_neutral_atoms; *E*^ext^ = 0) was attractive in the region 3.2 Å < *d* < 5.0 Å, and repulsive in the region 5.0 Å < *d* < 6.0 Å (Figure 7a). The maximum attractive force was −0.8 kcal/(mol Å) at the *d* = 3.8 Å position, and the maximum repulsive force was 0.4 kcal/(mol Å) at the *d* = 5.6 Å position (Figure 7b).

*E*_X_^ext^ = 100 MV/cm or *E*_Y_^ext^ = 100 MV/cm was applied to a water cluster containing two neutral solutes (Figure 7a); the mean force on *S*_2_, −*F*_X_(two_neutral_atoms; *E*^ext^), was computed using the trajectories of the MD simulations. The results showed that *F*_X_(two_neutral_atoms; *E*_X_^ext^ = 100 MV/cm) and *F*_X_(two_neutral_atoms; *E*_Y_^ext^ = 100 MV/cm) differed from *F*_X_(two_neutral_atoms; *E*^ext^ = 0). *F*_X_(two_neutral_atoms; *E*_X_^ext^ = 100 MV/cm) was larger than *F*_X_(two_neutral_atoms; *E*^ext^ = 0) in the region *d* < 4.4 Å, and less in the region 4.4 Å < *d* < 6.0 Å (Figure 7b). In contrast, *F*_X_(two_neutral_atoms; *E*_Y_^ext^ = 100 MV/cm) was similar to *F*_X_(two_neutral_atoms; *E*^ext^ = 0) in the region *d* < 4.4 Å, and *F*_X_(two_neutral_atoms; *E*_Y_^ext^ = 100 MV/cm) was larger than *F*_X_(two_neutral_atoms; *E*^ext^ = 0) in the region 4.4 Å < *d* < 6.0 Å (Figure 7b).

The PMF, −*P*_MF_(two_neutral_atoms; *E*^ext^), was calculated by integration of the mean force *F*_X_(two_neutral_atoms; *E*^ext^) from infinity. For comparison of the depths of the first wells of *P*_MF_(two_neutral_atoms; *E*^ext^) with *E*_X_^ext^ = 100 MV/cm or *E*_Y_^ext^ = 100 MV/cm, the second peak of *P*_MF_ was set to zero. The results showed that the depth of the first well of *P*_MF_(two_neutral_atoms; *E*^ext^ = 0) was smaller than that of *P*_MF_(two_neutral_atoms; *E*_X_^ext^ = 100 MV/cm), and similar to that of *P*_MF_(two_neutral_atoms; *E*_Y_^ext^ = 100 MV/cm) (Figure 7c).

For solutes *S*_1_ and *S*_2_ separated by a distance greater than 5.4 Å, the space between *S*_1_ and *S*_2_ can accommodate a water molecule (Figure 7d). When one of the water molecules, e.g., *W*_1_, stays at the position between solutes *S*_1_ and *S*_2_, *W*_1_ will push *S*_2_ along the +*x* direction. When *E*_X_^ext^ = 100 MV/cm is applied to this water cluster, the water molecules will be polarized along the *x* direction. *W*_1_ will be pushed by the neighboring water molecules, *W*_2_ and *W*_3_ (Figure 7e); the probability of one of the water molecules staying at the position of *W*_1_ when *E*_X_^ext^ = 100 MV/cm was applied was lower than that without application of an external electrostatic field. *F*_X_(two_neutral_atoms; *E*_X_^ext^ = 100 MV/cm) was therefore less than *F*_X_(two_neutral_atoms; *E*^ext^ = 0) in the region 4.4 Å < *d* < 6.0 Å (Figure 7b). If *E*_Y_^ext^ = 100 MV/cm is applied to this water cluster, the water molecules will be polarized along the *y* direction. *W*_1_ will be attracted by the neighboring water molecules, *W*_2_ and *W*_3_ (Figure 7f); the probability of one of the water molecules staying at the position of *W*_1_ under application of *E*_Y_^ext^ = 100 MV/cm was larger than that without application of an external electrostatic field. *F*_X_(two_neutral_atoms; *E*_Y_^ext^ = 100 MV/cm) was therefore larger than *F*_X_(two_neutral_atoms; *E*^ext^ = 0) in the region 4.4 Å < *d* < 6.0 Å (Figure 7b).

## 3. Method

### 3.1. MD Simulations

The simulations were performed in an NVE ensemble using the CHARMM package [39] and spherical boundary conditions. The ion-water and water-water interaction energies were calculated by summation of the electrostatic and vdW pairwise energies with a non-bond cutoff of 99 Å. For TIP3P water, the charge states of the oxygen and hydrogen atom were −0.834 e and +0.417 e, respectively, and the vdW parameters of the hydrogen atom were ɛ = −0.046 kcal/mol and *R*_min_/2 = 0.2245 Å. The O–H bond length of TIP3P, 0.9572 Å, and the bond angle of H–O–H, 104.52°, were constrained during the simulations using the SHAKE algorithm [43]. The intrinsic electronic polarizability of the water molecule changed as a strong electric field [44] was not considered in this project. All atoms were propagated according to Newton’s equations using the leapfrog Verlet algorithm and a time-step of 2 fs at a mean temperature of 300 K. Each system was first minimized for 1000 steps, equilibrated for 200 ps, and subsequently subjected to 1 ns of production. The configurations were stored every 20 fs.

### 3.2. Application of E^ext^ to Pure Water Cluster and Calculation of *p* and E^net^ from Trajectories of MD Simulations

*E*^ext^ was applied to a pure water cluster (Figure 1a), and the electric dipole moment per water molecule, *p*, was calculated using the sum of the electric dipole moments of water molecules with an oxygen atom distance origin ≤ *r*_cut_ over *N*_C_ configurations/frames, divided by the number of water molecules *N* with an oxygen atom distance origin ≤ *r*_cut_ over *N*_C_ configurations/frames:

(1) $$p=\frac{1}{N}\sum_{l=1}^{N_{\text{C}}}\sum_{m=1}^{n}\sum_{i=1}^{3}q_{\text{i}}r_{\text{i}}^{\text{lm}}u\left(r_{\text{cut}}-r_{\text{O}}^{\text{lm}}\right)$$

where *q*_i_ is the charge on water atom *i*, ***r***_i_^lm^ denotes the coordinates of atom *i* of water molecules *m* in configuration *l*, ***r***_O_^lm^ denotes the coordinates of the oxygen atom of water molecule *m* in configuration *l*, *n* is the number of solvent molecules in the simulation system, *N*c is the number of configurations/frames collected in equilibrium state in the MD simulations, and *u*(*r*_cut_ − *r*_O_^lm^) is the Heaviside unit step function.

*N* was computed as the sum of the water molecules with oxygen atoms positioned at distance origin ≤ *r*_cut_:

(2) $$N=\sum_{l=1}^{N_{\text{C}}}\sum_{m=1}^{n}u\left(r_{\text{cut}}-r_{\text{O}}^{\text{lm}}\right)$$

where the first summation is over *N*c configurations/frames, and the second summation is over the *n* solvent molecules in the simulation system.

The electrostatic field at the oxygen atom of TIP3P water, −***E***^net^, was contributed by ***E***^ext^ and water molecules with oxygen atom distance origins ≤ *r*_cut_ over *N*_C_ configurations/frames, divided by the number of water molecules *N* with oxygen atom distance origins ≤ *r*_cut_ over *N*_C_ configurations/frames as

(3) $$E^{\text{net}}=E^{\text{ext}}+\frac{1}{N}\sum_{l=1}^{N_{\text{C}}}\sum_{m^{\prime }=1}^{n}u\left(r_{\text{cut}}-r_{\text{O}}^{\text{l}{\text{m}}^{\prime }}\right)\sum_{\begin{array}{l} m=1\\ m^{\prime }\neq m \end{array}}^{n}\sum_{i=1}^{3}\frac{q_{\text{i}}}{4\pi {ɛ}_{0}{\left(R_{\text{iO}}^{\text{lm}{\text{m}}^{\prime }}\right)}^{3}}R_{\text{iO}}^{\text{lm}{\text{m}}^{\prime }}$$

where *ɛ*_0_ is the permittivity of free space, *q*_i_ is the charge on water atom *i*, ***R***^lmm′^_iO_ is the vector from atom *i* of water molecule *m* to the oxygen atom of water molecule *m*’ in configuration *l*.

### 3.3. For Charged Atom in Water Cluster, Calculation of *p*(*r*) and *E*^net^(*r*) from Trajectories of MD Simulations

For one charged atom in a water cluster (Figure 2a), *p*(*r*) was calculated by summing the electric dipole moments of water molecules with oxygen atoms located between (*r* − Δ*r*/2) and (*r* + Δ*r*/2) over *N*_C_ configurations/frames, divided by the number of water molecules *N*(*r*), as

(4) $$p(r)=\frac{1}{N(r)}\int_{r-\Delta r/2}^{r+\Delta r/2}\sum_{l=1}^{N_{\text{C}}}\sum_{m=1}^{n}\sum_{i=1}^{3}q_{i}\left(r_{\text{i}}^{\text{lm}}\times \frac{r_{\text{O}}^{\text{lm}}}{r_{\text{O}}^{\text{lm}}}\right)\delta \left(r^{\prime }-r_{\text{O}}^{\text{lm}}\right)\;dr^{\prime }$$

*N*(*r*) in (4) was computed as the sum of the number of water molecules whose oxygen atoms were at a distance from the solute of between (*r* − Δ*r*/2) and (*r* + Δ*r*/2):

(5) $$N(r)=\int_{r-\Delta r/2}^{r+\Delta r/2}\sum_{l=1}^{N_{\text{C}}}\sum_{m=1}^{n}\delta \left(r^{\prime }-r_{\text{O}}^{\text{lm}}\right)\;dr^{\prime }$$

where the first summation is over *N*c configurations/frames, the second summation is over the *n* solvent molecules in the simulation system, ***r***_O_^lm^ denotes the coordinates of the oxygen atom of water molecule *m* in configuration *l*, and Δ*r* is set at 0.1 Å.

*E*^net^(*r*) was calculated by summing the electrostatic fields of water with its oxygen atom located between (*r* − Δ*r*/2) and (*r +* Δ*r*/2) over *N*_C_ configurations/frames, divided by the number of water molecules *N*(*r*), as

(6) $$E^{\text{net}}(r)=\frac{1}{N(r)}\int_{r-\Delta r/2}^{r+\Delta r/2}\sum_{l=1}^{N_{\text{C}}}\sum_{m^{\prime }=1}^{n}\delta \left(r^{\prime }-r_{\text{O}}^{\text{l}{\text{m}}^{\prime }}\right)\left[\frac{q_{\text{j}}}{4\pi {ɛ}_{0}{\left(R_{\text{jo}}^{\text{l}{\text{m}}^{\prime }}\right)}^{2}}+\sum_{\begin{array}{l} m=1\\ m^{\prime }\neq m \end{array}}^{n}\sum_{i=1}^{3}\frac{q_{i}}{4\pi {ɛ}_{0}{\left(R_{\text{iO}}^{\text{lm}{\text{m}}^{\prime }}\right)}^{3}}\left(R_{\text{iO}}^{\text{lm}{\text{m}}^{\prime }}\times \frac{r_{\text{O}}^{\text{l}{\text{m}}^{\prime }}}{r_{\text{O}}^{\text{l}{\text{m}}^{\prime }}}\right)\right]dr^{\prime }$$

where *ɛ*_0_ is the permittivity of free space, *q*_i_ is the charge on water atom *i*, *q*_j_ is the charge on the solute atom, *R*^lm′^_jO_ is the distance between the oxygen atom of water molecule *m*’ in configuration *l* and solute *j*, ***R***^lmm′^_iO_ is the vector from atom *i* of water molecule *m* to the oxygen atom of water molecule *m*’ in configuration *l*.

### 3.4. Application of *E*^ext^ to Water Cluster Containing One or Two Solutes and Calculation of *F*^solv^(r) and *F*^net^(r) from Trajectories of MD Simulations

The net mean force on solute *S*_2_ with charge *Q*_2_, −*F*^net^, was decomposed and contributed by the external electrostatic field −*F*^E^, solute *S*_1_ with charge *Q*_1_, −*F*^S1^, and the polarized solvent molecules, −*F*^solv^. *F*^E^ was computed as *F*^E^ = *Q*_2_*E*.

*F*^S1^ was the force acting on solute *S*_2_ at *r*_2_ because of solute *S*_1_ at *r*_1_, and can be computed as the sum of the electrostatic and vdW forces as

(7) $$F_{\text{ele}}^{\text{S}1}=\frac{Q_{1}Q_{2}R_{12}}{4\pi {ɛ}_{0}{\left(R_{12}\right)}^{3}}$$

(8) $$F_{\text{vdw}}^{\text{S}1}=\frac{12{ɛ}_{12}}{R_{12}}\left[{\left(\frac{R_{\text{min},12}}{R_{12}}\right)}^{12}-{\left(\frac{R_{\text{min},12}}{R_{12}}\right)}^{6}\right]\frac{R_{12}}{R_{12}}$$

where *R*_12_ = *r*_2_ − *r*_1_, and the vdW parameters, ɛ_12_ and *R*_min,12_, were obtained using the standard combining rules.

*F*^solv^ was the force acting on solute *S*_2_ at *r*_2_ because of the solvent molecules, and can be computed as the sum of the electrostatic and vdW forces as

(9) $$F_{\text{ele}}^{\text{solvent}}=\frac{1}{N_{\text{C}}}\sum_{l=1}^{N_{\text{C}}}\sum_{m=1}^{n}\sum_{j=1}^{3}\frac{Q_{2}q_{j}}{4\pi {ɛ}_{0}{\left(R_{2\text{j}}^{\text{lm}}\right)}^{3}}R_{2\text{j}}^{\text{lm}}$$

(10) $$F_{\text{vdW}}^{\text{solvent}}=\frac{1}{N_{\text{C}}}\sum_{l=1}^{N_{\text{C}}}\sum_{m=1}^{n}\sum_{j=1}^{3}\frac{12{ɛ}_{2\text{j}}}{R_{2\text{j}}^{\text{lm}}}\left[{\left(\frac{R_{\text{min},2\text{j}}}{R_{2\text{j}}^{\text{lm}}}\right)}^{12}-{\left(\frac{R_{\text{min},2\text{j}}}{R_{2\text{j}}^{\text{lm}}}\right)}^{6}\right]\frac{R_{2\text{j}}^{\text{lm}}}{R_{2\text{j}}^{\text{lm}}}$$

where the first summation is over *N*c configurations/frames, the second summation is over the *n* solvent molecules in the simulation system, *q*_j_ is the charge on water atom *j*, *R*^lm^_2j_ is the vector from atom *j* of water molecule *m* to solute *S*_2_ at *r*_2_ in configuration *l*, and the vdW parameters, ɛ_2j_ and *R*_min,2j_, were obtained using the standard combining rules.

## 4. Conclusions

To explore the athermal effect of ***E***^ext^ on the stabilities of protein conformations and the binding affinities of protein-protein/ligand interactions, the dependence of the mean force between charged solutes or neutral solutes, *S*_1_ and *S*_2_, on ***E***^ext^ was studied using MD simulations. The results showed that (1) ***E***^ext^ shifts the equilibrium position of the *p* − *E*^net^ curve of the water molecule, and may reduce the dielectric shielding effect between charged atoms in macromolecules; (2) For ***E***^ext^ along the direction of the electric dipole formed by *S*_1_ and *S*_2_, *E*^ext^ = 40 or 60 MV/cm enhances the polar interactions between the two charged solutes; (3) For ***E***^ext^ perpendicular to the direction of the electric dipole formed by *S*_1_ and *S*_2_, *E*^ext^ = 100 MV/cm enhances the polar interactions between these two charged solutes; (4) The mean force and the PMF between two neutral solutes depend on ***E***^ext^.
